# Heart Rate Variability's Value in Predicting Out-of-Hospital Major Adverse Cardiovascular Events in Patients With Chronic Heart Failure

**DOI:** 10.1155/cdr/6412775

**Published:** 2025-08-14

**Authors:** Li Men, Bingxin Chen, Long Yang, Jiangrong Shi, Shuqin Tang, Xing Jiang, Yunhua Chen, Xiao Wang, Ping Fan

**Affiliations:** ^1^Department of Heart Function, First Affiliated Hospital of Xinjiang Medical University, Urumqi, Xinjiang, China; ^2^Department of Pediatric Cardiothoracic Surgery, First Affiliated Hospital of Xinjiang Medical University, Urumqi, Xinjiang, China; ^3^Department of Neonatology, First Affiliated Hospital of Xinjiang Medical University, Urumqi, Xinjiang, China; ^4^Electrocardiogram Room, Shawan People's Hospital, Tacheng Region, Xinjiang, China; ^5^Department of Cardiovascular Medicine, Kucha People's Hospital, Aksu Region, Xinjiang, China; ^6^Electrocardiogram Room, Huo County People's Hospital, Yili Kazakh Autonomous Prefecture, Xinjiang, China; ^7^Electrocardiogram Room, Shache County People's Hospital, Kashgar Region, Xinjiang, China

**Keywords:** chronic heart failure, cohort study, heart rate variability, Holter, MACEs

## Abstract

**Background:** Chronic heart failure (CHF) involves changes in cardiac structure and function, along with extensive neuroendocrine adaptations and metabolic abnormalities. Heart rate variability (HRV) is a noninvasive measure of autonomic nervous system function and is associated with mortality in CHF. However, the significance of HRV in predicting major adverse cardiovascular events (MACEs) in CHF patients has not been fully explored. This study was aimed at investigating the predictive value of HRV parameters assessed by 24-h Holter monitoring for MACEs in CHF patients.

**Methods:** This prospective cohort study included 906 CHF patients from five centers in Xinjiang, China, who underwent Holter monitoring and were followed up. Cox proportional hazards regression models were used to assess the independent associations between HRV parameters and the incidence of MACEs. Receiver operating characteristic (ROC) curve analysis was conducted to determine the predictive accuracy of each HRV parameter, and the incremental predictive value of HRV parameters was evaluated using coherence index (*C*-index), net reclassification improvement (NRI), and integrated discrimination improvement (IDI).

**Results:** During a median follow-up of 16 months, 211 (23.3%) MACEs occurred. Cox regression analysis indicated that SDNN (HR: 0.976, 95% CI: 0.970~0.981), triangular index (HR: 0.963, 95% CI: 0.953~0.973), SDNN index (HR: 0.983, 95% CI: 0.974~0.992), SDANN index (HR: 0.974, 95% CI: 0.967~0.981), NN50 (HR: 0.859, 95% CI: 0.787~0.937), rMSSD (HR: 0.980, 95% CI: 0.970~0.989), TP (HR: 0.890, 95% CI: 0.816~0.971), VLF (HR: 0.889, 95% CI: 0.815~0.969), LF (HR: 0.817, 95% CI: 0.743~0.898), and HF (HR: 0.806, 95% CI: 0.728~0.893) were independently associated with MACEs. ROC analysis revealed that the triangular index and SDNN had the highest area under the curve (AUC) for predicting MACEs, with values of 0.699 (95% CI: 0.655~0.743) and 0.711 (95% CI: 0.668~0.753), respectively. Incorporation of HRV parameters into traditional risk models improves the *C*-index, NRI, and IDI of the model's predictive ability for MACE and cardiovascular mortality to varying degrees.

**Conclusion:** SDNN and triangular index demonstrated the strongest predictive abilities; other time–domain and frequency–domain parameters also showed certain predictive values for MACEs.

## 1. Background

Chronic heart failure (CHF) is a common progressive condition characterized by the heart's inability to pump sufficient blood to meet the body's metabolic demands [1]. It is estimated that the global prevalence among the general adult population is 1%–2%, with rates exceeding 10% in individuals aged 70 and older [2]. In China, the prevalence of CHF is estimated to be approximately 1.3%, affecting about 13.7 million people, with a particularly heavy burden in rural areas and among the elderly. In Xinjiang, the unique geographic features, dietary habits, and limited healthcare resources further exacerbate the burden of CHF [3, 4]. Despite advances in treatment strategies, CHF patients continue to face a high risk of cardiovascular adverse events (MACEs), such as cardiovascular death, acute myocardial infarction, and stroke [5, 6]. These events often lead to reduced quality of life and increased healthcare utilization, highlighting the urgent need for effective risk stratification and early intervention strategies.

The cardiovascular system is regulated by the autonomic nervous system (ANS), and dysfunction of the ANS can exacerbate neurohormonal activation, impair cardiac output, and promote arrhythmias, leading to the worsening of CHF [7]. To assess ANS activity, heart rate variability (HRV) has been proposed as a promising noninvasive biomarker. HRV reflects the physiological fluctuations in the time intervals between consecutive heartbeats and is influenced by the dynamic interplay between the sympathetic and parasympathetic nervous system inputs [8]. The prognostic value of HRV lies in its ability to provide insights into autonomic dysfunction. Sympathetic hyperactivity and reduced parasympathetic tone, leading to autonomic imbalance, are key factors contributing to adverse remodeling, arrhythmias, and hemodynamic instability in CHF. HRV parameters such as time–domain and frequency–domain measurements offer quantitative indicators to assess this imbalance [9]. Several studies have shown that reduced HRV is associated with increased morbidity and mortality in patients with coronary artery disease, myocardial infarction, and heart failure [10–12].

Gatzoulis et al. found that HRV can predict the risk of arrhythmia in patients with myocardial infarction within 32 months [13]. Two studies further indicated that short-term HRV can predict the risk of death in patients with heart failure and after transcatheter aortic valve replacement (TAVR) [14, 15]. Previous studies have shown that HRV has a strong predictive ability for out-of-hospital MACEs in patients with acute ST-elevation myocardial infarction (STEMI) undergoing percutaneous coronary intervention (PCI) [16]. Current research on CHF has primarily focused on short-term HRV measurements and mortality outcomes, while MACEs, which are more representative of long-term real-world outcomes, have not received sufficient attention. This study is aimed at evaluating the predictive value of HRV parameters derived from 24-h dynamic electrocardiogram (ECG) (Holter) monitoring in forecasting out-of-hospital MACEs in patients with CHF. This research seeks to provide new insights into the prognostic utility of HRV and its integration into clinical practice.

## 2. Methods

### 2.1. Study Design

This multicenter prospective cohort study is aimed at evaluating the predictive value of 24-h Holter monitoring parameters in forecasting out-of-hospital MACEs in patients with CHF. The research team recruited CHF patients diagnosed and treated at the First Affiliated Hospital of Xinjiang Medical University and its collaborating centers from January 2022 to June 2023. The collaborating centers include Huo County People's Hospital (Yili Kazakh Autonomous Prefecture, Xinjiang, China), Kucha People's Hospital (Aksu Region, Xinjiang, China), Shawan People's Hospital (Tacheng Region, Xinjiang, China), and Shache County People's Hospital (Kashgar Region, Xinjiang, China). Patients were enrolled in the study when they visited the cardiovascular department due to worsening symptoms of heart failure, treatment of other comorbidities, adjustment of drug therapy, or assessment of treatment efficacy. This study was conducted according to the principles of the Declaration of Helsinki, and the research protocol was approved by the Human Ethics Committee of the First Affiliated Hospital of Xinjiang Medical University (Approval Number K202309-12-2409A-Y1). All patients involved in the study provided written informed consent.

### 2.2. Study Population

This study will include patients who meet the following criteria: (1) age ≥ 18 years, regardless of sex; (2) diagnosed with CHF according to the China Heart Failure Diagnosis and Treatment Guidelines 2018 [4], based on the following criteria: patients have relevant medical history, such as coronary artery disease, hypertension, heart failure risk factors, or symptoms like orthopnea or paroxysmal nocturnal dyspnea; physical examination reveals signs such as lung rales, bilateral lower limb edema, heart murmurs, jugular venous distension, displaced or diffuse apical impulse; chest x-ray shows pulmonary congestion, pulmonary edema, or cardiomegaly; ECG results show previous myocardial infarction, atrial fibrillation (AF), or conduction abnormalities; echocardiography reveals structural and/or functional heart abnormalities; laboratory tests show elevated biomarkers such as B-type natriuretic peptide (BNP) or N-terminal pro-B-type natriuretic peptide (NT-proBNP); (3) able to complete 24-h Holter monitoring and willing to participate in long-term follow-up; and (4) provided written informed consent.

Patients who meet any of the following criteria will be excluded from the study: (1) mental illness or psychological disorders; (2) severe liver dysfunction (including significant elevation of alanine aminotransferase [ALT] or aspartate aminotransferase [AST]); (3) other severe cardiac diseases, such as severe valvular heart disease, hypertrophic cardiomyopathy, or congenital heart disease; (4) severe arrhythmias; (5) unable to undergo Holter monitoring or unable to wear the Holter monitor for less than 6 h; (6) severe genetic disorders; and (7) refusal to participate in the study.

### 2.3. Clinical Data Collection

Clinical data were collected by trained cardiovascular specialists. Upon admission, data such as age, sex, medical history, smoking history, alcohol consumption, and New York Heart Association (NYHA) classification were recorded. Physical and laboratory examinations were performed by trained nurses and included measurements of the patient's height, weight, and resting blood pressure. On admission, 8 mL of venous blood was drawn to evaluate markers including white blood cell (WBC) count, red blood cell (RBC) count, hemoglobin (HB), C-reactive protein (CRP), interleukin-6 (IL-6), NT-proBNP, and estimated glomerular filtration rate (eGFR) [17]. Within 24 h of admission, 4 mL of fasting venous blood was collected from each patient to assess fasting blood glucose (FBG), hemoglobin A1c (HbA1c), triglycerides (TG), total cholesterol (TC), high-density lipoprotein cholesterol (HDL-C), and low-density lipoprotein cholesterol (LDL-C). Echocardiography was performed using a VIVID7 color Doppler ultrasound diagnostic system (GE Healthcare, United States) to measure left ventricular ejection fraction (LVEF). Data from the collaborating centers were also collected in the same manner and stored in the medical record system and were reviewed and organized by trained physicians from the First Affiliated Hospital of Xinjiang Medical University.

### 2.4. Twenty-Four–Hour Holter Monitoring

Within 24 h of admission, 24-h Holter monitoring was performed using a device (MedEx MECG-200, Beijing, China). During the monitoring period, patients were instructed to maintain their usual daily activities to ensure that HRV data accurately reflected ANS function in real-life conditions. After 24 h of continuous monitoring, the device was retrieved, and the data were analyzed using MedEx software. Cardiologists from all four collaborating centers received uniform training from our hospital and used the same method to monitor and analyze 24-h Holter data in CHF patients. The following parameters were recorded in this study: maximum heart rate, minimum heart rate, average heart rate, and HRV-related parameters.

Time–domain analysis primarily relied on statistical analysis of NN intervals (or RR intervals, referred to as NN intervals in this paper) in the 24-h Holter records. The following parameters were recorded in this study: standard deviation of NN intervals (SDNN, millisecond); triangular index; SDNN index (millisecond), representing the average standard deviation of NN intervals within a 5-min time window; SDANN index (millisecond), representing the standard deviation of average NN intervals within a 24-h time window; count of consecutive NN intervals differing by more than 50 ms (NN50); root mean square of successive differences (rMSSD, millisecond); percentage of consecutive NN intervals differing by more than 50 ms (pNN50, percentage).

The frequency–domain analysis involved power spectral analysis (fast Fourier transform model) of the heart rate sequence, breaking total power (TP) into different frequency bands. The following parameters were recorded in this study: TP (ms^2^), representing the TP of NN intervals across the entire frequency spectrum (0–0.4 Hz); ultralow frequency (ULF, ms^2^), representing the power in the 0–0.0033 Hz range; very low frequency (VLF, ms^2^), representing the power in the 0.0033–0.04 Hz range; low frequency (LF, ms^2^), representing the power in the 0.04–0.15 Hz range; high frequency (HF, ms^2^), representing the power in the 0.15–0.4 Hz range; the ratio of LF power to HF power (LF:HF).

### 2.5. Follow-Up Assessment

From the date of admission to discharge, patients will participate in a follow-up program lasting 18 months, with the overall follow-up completion date set for December 20, 2024. We collected patient contact information and conducted follow-up through telephone calls and outpatient visits. The follow-up work is carried out by trained cardiovascular specialists from the First Affiliated Hospital of Xinjiang Medical University, including patients from the collaborating centers. The primary purpose of follow-up is to record MACEs, under the ACC/AHA clinical trial center cardiovascular endpoint event recommendations, including nonfatal myocardial infarction, cardiogenic shock, malignant arrhythmia, unstable angina, stroke, hospitalization due to worsening heart failure, and cardiovascular death [18]. During the follow-up, the occurrence of any of these events is considered a MACE, and follow-up is terminated. Multiple MACEs can occur in a patient at the same time.

### 2.6. Statistical Analysis

All statistical analyses in this study were performed using R software (Version 4.4.2), with a two-sided *p* value < 0.05 considered statistically significant. Participants were divided into MACE and non-MACE groups based on the occurrence of MACEs, and baseline characteristics and HRV parameters of CHF patients were compared. Continuous variables with nonnormal distribution were expressed as the median and interquartile range (25th and 75th percentiles), and intergroup comparisons were conducted using the Mann–Whitney *U* test. Categorical variables were expressed as frequencies (percentages), and intergroup comparisons were made using the chi-square test. Spearman correlation analysis was used to construct heatmaps to explore the associations between HRV parameters and cardiovascular risk factors. CHF patients were further grouped based on the tertiles of each HRV parameter, and Kaplan–Meier survival curves were used to analyze survival rates by HRV levels, with log-rank tests comparing survival rate differences between groups. Cox proportional hazards regression models were used to investigate the associations between HRV parameters and out-of-hospital MACEs in CHF patients. The baseline model did not adjust for any covariates; Model 1 adjusted for patient demographics, including sex, age, BMI, smoking status, drinking status, hypertension, and NYHA classification; Model 2 further adjusted for laboratory test indicators, including lymphocyte count, eGFR, NT-proBNP, albumin, HbA1c, TG, TC, HDL-C, LDL-C, and IL-6. The predictive ability of various indicators for the occurrence of MACE in STEMI patients was evaluated using receiver operating characteristic (ROC) curves. The improvement in model predictive ability after adding HRV indicators to the traditional risk factor model was assessed using the concordance index (*C*-index), net reclassification improvement (NRI), and integrated discrimination improvement (IDI) [19].

## 3. Results

### 3.1. Baseline Characteristics of Patients With CHF

**Figure 1:**
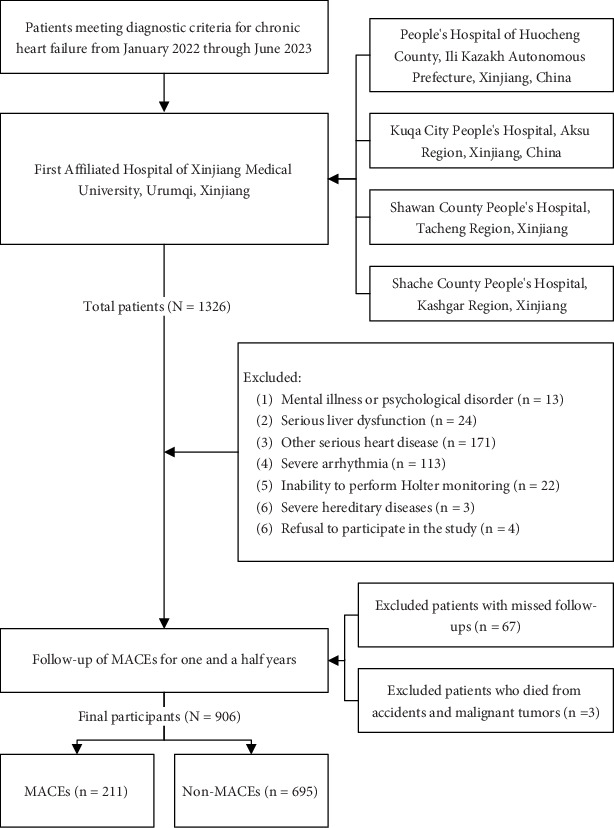
Flowchart for inclusion–exclusion of study design.

A total of 1326 CHF patients from the First Affiliated Hospital of Xinjiang Medical University and its four collaborating centers were enrolled in this study. After initial screening, 350 patients who did not meet the study criteria were excluded, leaving 976 patients for the follow-up study. During the follow-up period, 67 patients (6.8%) withdrew for various reasons, including 1 patient who died in a traffic accident and 2 patients who died of malignancies. Ultimately, 906 participants who met the study criteria were included, consisting of 743 (82.0%) from the First Affiliated Hospital of Xinjiang Medical University, 49 (5.4%) from Kucha People's Hospital, 44 (4.9%) from Shawan People's Hospital, 29 (3.2%) from Shache People's Hospital, and 41 (4.5%) from Huo County People's Hospital. During the follow-up period, 211 patients experienced MACEs (Figure 1).

The median age of CHF patients in this study was 61 years, including 302 females (33.3%) and 604 males (66.7%). Patients were further categorized into MACE and non-MACE groups based on the occurrence of MACEs; their baseline characteristics were compared (Table 1). The results showed that patients in the MACE group were older and had a higher smoking rate, a higher prevalence of hypertension, and a higher proportion in NYHA Class III and IV (*p* < 0.05). Additionally, there were significant statistical differences between the two groups in BMI, drinking status, lymphocyte count, NT-proBNP, eGFR, HbA1c, TG, TC, HDL-C, LDL-C, albumin, and IL-6 levels (*p* < 0.05). No significant statistical differences were observed between the two groups in terms of gender, blood pressure, diabetes, coronary heart disease (CHD), WBC, neutrophils, lymphocytes, erythrocytes, HB, FBG, CRP, and LVEF levels (*p* > 0.05).

### 3.2. Characteristics of HRV Monitored by 24-h Holter Patients With CHF

Regarding the overall heart rate measurements, the average heart rate and minimum heart rate in the MACE group were significantly higher than those in the non-MACE group (*p* < 0.05). The difference in maximum heart rate between the two groups was minor but statistically significant (*p* = 0.035). Analysis of the HRV time–domain parameters showed significant differences between the two groups. Compared to the non-MACE group, the MACE group had significantly lower SDNN, triangular index, SDNN index, SDANN index, NN50, and rMSSD (*p* < 0.05), while no significant difference was observed in pNN50 (*p* > 0.05). In the frequency-domain analysis, significant differences between the groups were also observed. The MACE group had significantly lower TP, ULF, VLF, LF, and HF compared to the non-MACE group (*p* < 0.05). There was no significant difference in the LF/HF ratio between the two groups (*p* > 0.05) (Table 2).

### 3.3. Correlation Analysis of HRV Indices With Cardiac Risk Factors

**Figure 2:**
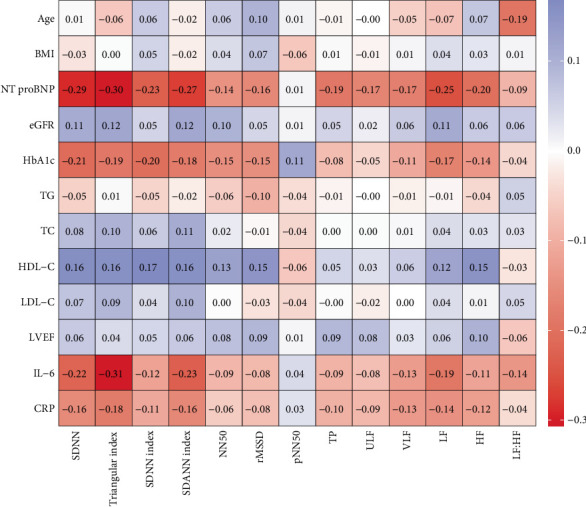
Heat map of the correlation between HRV indices and cardiovascular risk factors in patients with CHF. Note: A blue grid indicates a positive correlation and a red grid indicates a negative correlation, with darker colors representing higher correlations.

Spearman correlation analysis revealed that HRV indices were associated with multiple cardiac risk factors (Figure 2). Time–domain analysis parameters such as SDNN, triangular index, SDNN index, SDANN index, NN50, and rMSSD were correlated with NT-proBNP, HbA1C, and HDL-C (*p* < 0.05). Frequency–domain analysis parameters, including TP, VLF, LF, and HF, were correlated with NT-proBNP and HbA1C (*p* < 0.05). Age was correlated with LF, HF, LF:HF ratio, and rMSSD (*p* < 0.05). HRV indices showed a certain degree of correlation with various cardiac risk factors, and the specific correlation coefficients and *p* values can be found in Supporting Information 3: Table S1.

### 3.4. Relationship Between HRV and the Incidence of Out-of-Hospital MACEs in Patients With CHF

During a median follow-up of 16 months, 211 MACEs occurred, with patients experiencing multiple MACEs simultaneously. These included malignant arrhythmias in 10 (4.7%) patients, unstable angina in 68 (32.2%) patients, nonfatal myocardial infarction in 17 (8.1%) patients, cardiogenic shock in 37 (17.5%) patients, stroke in 10 (4.7%) patients, heart failure exacerbation leading to hospitalization in 121 (57.3%) patients, and cardiovascular death in 36 (17.1%) patients.

**Figure 3:**
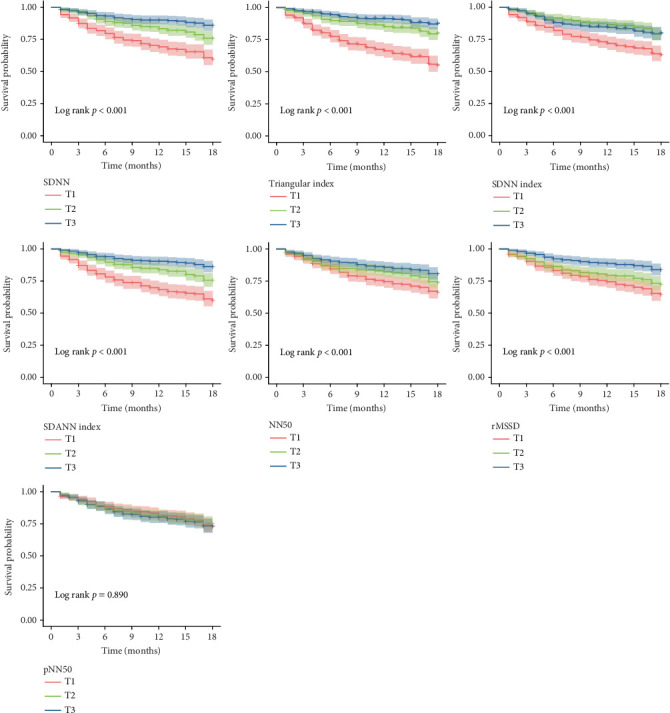
Survival curves for the incidence of MACEs in CHF patients grouped by tertiles of each HRV time–domain analysis metric.

**Figure 4:**
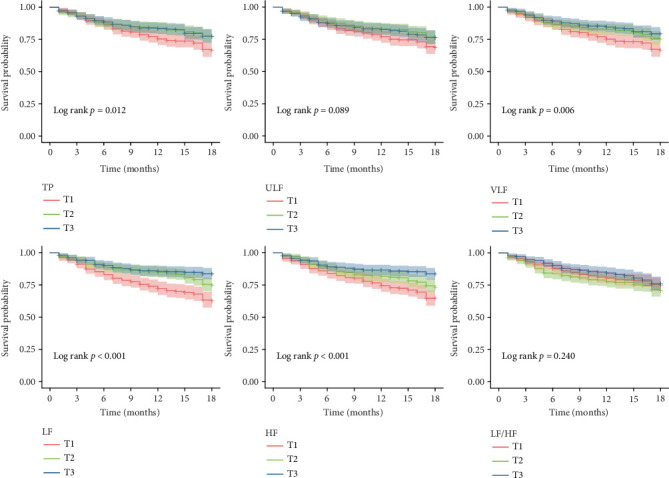
Survival curves for the incidence of MACEs in CHF patients grouped by tertiles of each HRV frequency–domain analysis metric.

Based on the tertiles of each HRV parameter, CHF patients were grouped, and Kaplan–Meier survival curves were plotted. The results indicated that for time–domain analysis parameters, the first tertile (T1) group for SDNN, triangular index, SDNN index, SDANN index, NN50, and rMSSD had the lowest survival rates for MACEs (log-rank *p* < 0.05) (Figure 3). In frequency–domain analysis, the T1 group for TP, VLF, LF, and HF also had the lowest survival rates for MACEs (log-rank *p* < 0.05) (Figure 4). In the analysis of cardiovascular mortality, the T1 group for time–domain indicators, including SDNN, triangular index, SDNN index, SDANN index, and rMSSD, was associated with an increased cardiovascular mortality rate in CHF patients (log-rank *p* < 0.05) (Supporting Information 1: Figure S1). Additionally, for frequency–domain analysis parameters, no significant differences were observed among the groups for cardiovascular mortality (log-rank *p* > 0.05) (Supporting Information 2: Figure S2).

This study further conducted a Cox proportional hazards regression analysis to explore the relationship between HRV indices and the incidence of MACEs (Table 3). The results indicated that in the unadjusted model, SDNN, triangular index, SDNN index, SDANN index, NN50, rMSSD, TP, ULF, VLF, LF, and HF were significantly associated with MACEs (*p* < 0.05). After adjusting for the general characteristics of the patients in Model 1, these indices continued to show an independent association with MACEs. Further adjustment for laboratory indices in Model 2 revealed that SDNN (HR: 0.976, 95% CI: 0.970~0.981, *p* < 0.001), triangular index (HR: 0.963, 95% CI: 0.953~0.973, *p* < 0.001), SDNN index (HR: 0.983, 95% CI: 0.974~0.992, *p* < 0.001), SDANN index (HR: 0.974, 95% CI: 0.967~0.981, *p* < 0.001), NN50 (HR: 0.859, 95% CI: 0.787~0.937, *p* < 0.001), rMSSD (HR: 0.980, 95% CI: 0.970~0.989, *p* < 0.001), TP (HR: 0.890, 95% CI: 0.816~0.971, *p* = 0.009), VLF (HR: 0.889, 95% CI: 0.815~0.969, *p* = 0.007), LF (HR: 0.817, 95% CI: 0.743~0.898, *p* < 0.001), and HF (HR: 0.806, 95% CI: 0.728~0.893, *p* < 0.001) were independently associated with MACEs. However, pNN50 (HR: 0.996, 95% CI: 0.990~1.002, *p* = 0.186), ULF (HR: 0.945, 95% CI: 0.888~1.006, *p* = 0.079), and LF/HF (HR: 0.981, 95% CI: 0.936~1.028, *p* = 0.426) were not significantly associated with MACEs.

This study also performed a Cox proportional hazards regression analysis to examine the relationship between HRV indices and cardiovascular mortality in CHF patients (Table 4). The results showed that in the unadjusted model, SDNN, triangular index, SDANN index, rMSSD, TP, ULF, VLF, LF, and HF were associated with cardiovascular mortality (*p* < 0.05). In Model 1, the relationship between VLF and HF with mortality was no longer significant. In the final Model 2, only frequency–domain indicators, including SDNN (HR: 0.973, 95% CI: 0.958~0.988, *p* < 0.001), triangular index (HR: 0.913, 95% CI: 0.879~0.947, *p* < 0.001), SDANN index (HR: 0.969, 95% CI: 0.951~0.987, *p* = 0.001), and rMSSD (HR: 0.970, 95% CI: 0.942~0.998, *p* = 0.036) were independently associated with cardiovascular mortality. In contrast, time–domain analysis parameters such as TP, ULF, VLF, LF, and HF were no longer significantly associated with mortality. Furthermore, in all three models, the SDANN index, pNN50, and LF/HF did not show a significant association with cardiovascular mortality.

### 3.5. Predictive Value of Each HRV Parameter for MACEs and Cardiovascular Mortality in CHF Patients

**Figure 5:**
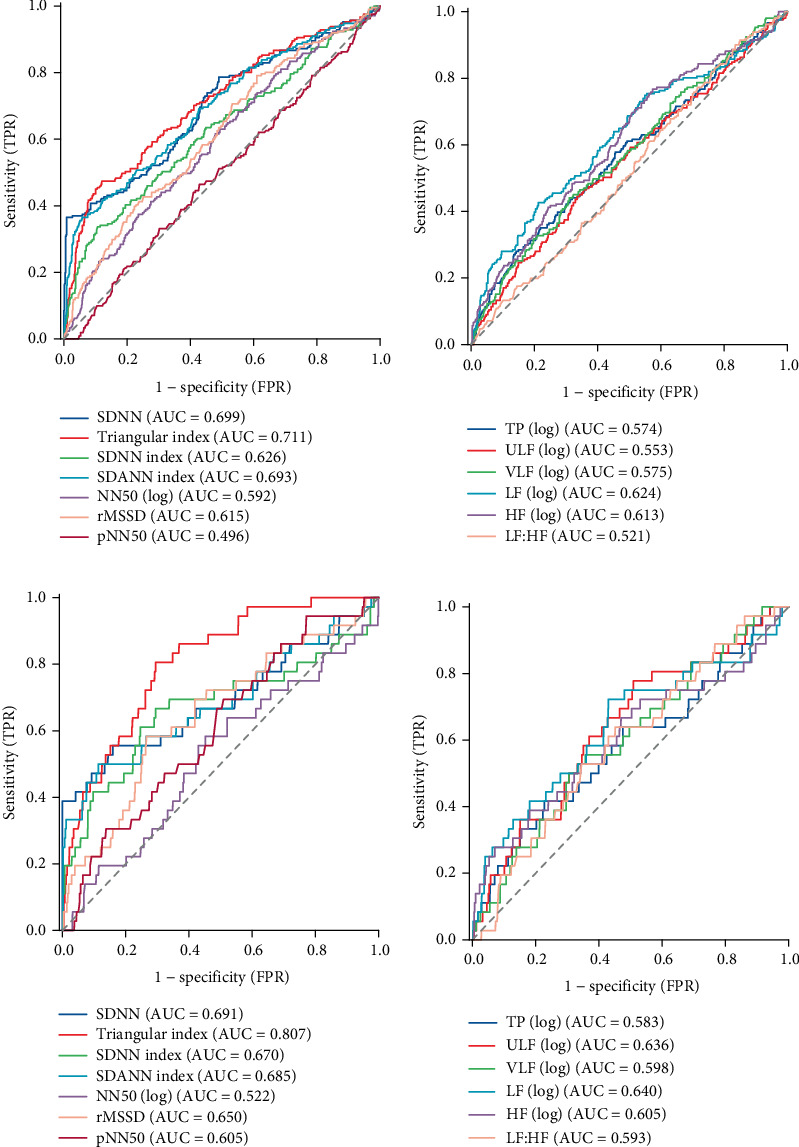
ROC curves for HRV metrics to predict the incidence of MACEs and cardiovascular death in patients with CHF. (a) Time–domain analysis metrics predicting MACEs. (b) Frequency–domain metrics predicting MACEs. (c) Time–domain analysis metrics predicting cardiovascular death. (d) Frequency–domain metrics predicting cardiovascular death.

This study used ROC analysis to investigate the individual predictive ability of each HRV parameter for MACEs. The results showed that time–domain analysis parameters, including the triangular index (AUC: 0.711, 95% CI: 0.668–0.753), SDNN (AUC: 0.699, 95% CI: 0.655–0.743), and SDANN index (AUC: 0.693, 95% CI: 0.650–0.737), demonstrated good predictive ability, while NN50, rMSSD, and pNN50 showed weaker predictive ability (Figure 5a). In frequency–domain analysis, LF (AUC: 0.624, 95% CI: 0.578–0.670) and HF (AUC: 0.613, 95% CI: 0.569–0.657) showed moderate predictive ability. TP, ULF, and VLF exhibited poor predictive capacity, and the LF/HF ratio (AUC: 0.521, 95% CI: 0.477–0.564) showed the weakest predictive ability, close to random chance (Figure 5b). Additionally, SDNN, triangular index, SDNN index, SDANN index, and TP displayed good specificity, while LF/HF showed good sensitivity. SDNN had the highest Youden index (Table 5).

In predicting cardiovascular mortality, the triangular index (AUC: 0.807, 95% CI: 0.741–0.874) demonstrated the best discriminatory ability. SDNN (AUC: 0.691, 95% CI: 0.577–0.804), SDNN index (AUC: 0.670, 95% CI: 0.556–0.784), SDANN index (AUC: 0.685, 95% CI: 0.575–0.795), and rMSSD (AUC: 0.650, 95% CI: 0.552–0.748) showed moderate predictive ability. NN50 and pNN50 exhibited weak predictive power (Figure 5c). Among frequency–domain parameters, LF (AUC: 0.640, 95% CI: 0.534–0.746) and ULF (AUC: 0.636, 95% CI: 0.541–0.730) demonstrated moderate predictive ability, while TP, VLF, HF, and LF/HF showed poor predictive power (Figure 5d). SDNN, SDANN index, and HF displayed good specificity, while the triangular index demonstrated good sensitivity and had the highest Youden index (Supporting Information 3: Table S2).

This study further constructed an initial model based on basic patient information and laboratory tests of CHF patients. HRV parameters were added to the original model, and the *C*-index was used to observe the probability that the model's predictions matched the actual outcomes. NRI and IDI were used to assess the improvement in the model's predictive ability after adding HRV parameters. The results indicated that, in predicting MACEs, the triangular index and SDNN were the best predictors for CHF patients, as evidenced by higher *C*-index values and significant improvements in IDI and NRI. The SDANN index and rMSSD also demonstrated good predictive ability, contributing significantly to discrimination and reclassification. However, the improvement in predictive ability from frequency–domain analysis parameters was modest (Table 6).

The triangular index showed exceptional predictive value for predicting cardiovascular mortality in CHF patients. The *C*-index was 0.891 (95% CI: 0.851–0.930), IDI was 0.087 (95% CI: 0.035–0.205), and NRI was 0.420 (95% CI: 0.192–0.581). In contrast, other HRV parameters demonstrated only moderate value (Supporting Information 3: Table S3).

## 4. Discussion

This study primarily investigated the clinical value of HRV parameters in predicting MACEs and cardiovascular mortality in CHF patients. The results indicated that patients who experienced MACEs had higher cardiovascular risk factor levels and lower HRV levels compared to the non-MACE group. In time–domain analysis, SDNN, triangular index, SDNN index, SDANN index, NN50, and rMSSD were significantly lower in the MACE group than in the non-MACE group. Similarly, frequency–domain parameters including TP, ULF, VLF, LF, and HF also showed significant differences between the two groups. These HRV parameters were found to be correlated with cardiac risk factors such as NT-proBNP, HbA1c, and HDL-C. In Cox proportional hazards regression analysis, several HRV indices demonstrated independent associations with MACEs and cardiovascular mortality. Triangular index and SDNN showed higher AUC values in predicting MACEs, while triangular index exhibited the best predictive ability for cardiovascular mortality. Furthermore, adding HRV parameters to the traditional models improved the predictive ability for MACEs and cardiovascular mortality.

The findings of this study are consistent with existing literature. Previous research has shown that reduced HRV is associated with increased mortality and adverse cardiovascular events in various cardiac populations. For example, studies by Compostella and Coviello confirmed that lower time–domain HRV parameters such as SDNN and LF can predict higher mortality and MACEs in patients following myocardial infarction [20, 21]. Research by Bruno and Larisa also demonstrated that HRV parameters like SDNN, sNN50, rMSSD, and LF are associated with cardiovascular mortality in CHF patients [14, 22]. This study identified out-of-hospital MACEs in CHF patients as the primary outcome, providing practical significance for long-term clinical management and outpatient monitoring strategies. Previous studies have mainly emphasized hospitalization events or all-cause mortality. The results of this study reinforce the potential usefulness of HRV as a routine outpatient follow-up prediction tool, especially in areas with limited medical services such as Xinjiang.

CHF involves structural and functional changes in the heart itself and encompasses widespread neuroendocrine adaptation and metabolic abnormalities. CHF is typically associated with persistent sympathetic nervous system activation, which increases myocardial oxygen consumption, exacerbates cardiac load, accelerates cardiac remodeling, and may ultimately worsen the condition [23, 24]. In this study, HRV was found to be correlated with NT-proBNP, HbA1c, HDL-C, and inflammation. NT-proBNP is a classic biomarker of heart failure, primarily secreted by ventricular myocytes in response to excessive load or ventricular dilation. Elevated NT-proBNP levels typically indicate enhanced cardiac stress, particularly increased ventricular pressure and reduced cardiac pumping function. In CHF patients, the level of NT-proBNP is negatively correlated with HRV, suggesting a gradual loss of autonomic regulation in heart failure patients [25]. HbA1c reflects the average blood glucose levels over the past few months and is commonly used to assess the control of diabetes. Poor blood sugar control is often associated with autonomic dysfunction [26]. HDL-C is commonly known as “good” cholesterol and has anti-inflammatory, antioxidant, and vascular protective effects [27]. The relationship between decreased HRV and lower HDL-C levels suggests that autonomic dysfunction interacts with cardiac metabolic imbalance, inflammatory responses, and oxidative stress [28]. Furthermore, CHF patients often experience a state of chronic low-grade inflammation, with elevated levels of CRP, IL-6, and other markers, which further promote cardiac remodeling and fibrosis [29].

This study also indicates that in time–domain analysis, SDNN, triangular index, SDNN index, and SDANN index have higher predictive specificity, with triangular index and SDNN being the best predictors for CHF patients. SDNN comprehensively reflects the activity of both the sympathetic and parasympathetic nervous systems, with higher SDNN values typically indicating better cardiac autonomic regulation and lower cardiovascular risk [30]. SDNN index and SDANN index reflect HRV over shorter (e.g., 5 min) and longer (e.g., 24 h) time windows, respectively. These indices capture autonomic regulation changes at different time scales, providing more detailed information on cardiac regulation [31]. The SDANN index particularly reflects the stability of long-term HRV, which is closely related to the heart's ability to adapt to long-term stress [32]. The triangular index, which is the ratio of the area under the histogram of all NN intervals (normal sinus beat intervals) to the maximum height of the histogram base, provides a comprehensive reflection of the overall distribution and variability of HRV [33]. NN50 reflects the number of consecutive heartbeats that differ by more than 50 ms, indicating the heart's responsiveness to rapid autonomic input changes. rMSSD is sensitive to short-term HRV [34]. Since MACEs refer to a composite of various cardiovascular events, this explains why the triangular index and SDNN, both of which reflect the overall distribution of autonomic function, have strong predictive ability. Additionally, this study found that the triangular index showed good predictive ability for cardiovascular mortality. From a physiological perspective, the triangular index reflects the total variability of heart rate, integrating the effects of the sympathetic and parasympathetic nervous systems throughout the entire circadian cycle [35, 36]. A higher triangular index typically indicates preserved ANS flexibility, while a lower triangular index suggests blunted or dysregulated ANS responses, which are markers of CHF progression. A reduced triangular index is associated with increased sympathetic tone, impaired baroreflex sensitivity, and an increased risk of arrhythmias, all of which contribute to the pathophysiology of adverse cardiovascular outcomes [37]. Previous studies have established the triangular index as a reliable predictor of cardiovascular disease prognosis. For example, Zhang et al. demonstrated that a reduced triangular index predicts MACEs after myocardial infarction [38]. Hämmerle et al. found that the triangular index obtained from a 5-min ECG recording has the ability to accurately predict cardiovascular mortality risk in patients with AF [39]. The superior performance of the triangular index in this cohort may be attributed to its robustness to signal-to-noise ratios and its comprehensive reflection of long-term ANS activity, which aligns with the chronic and dynamic nature of heart failure.

Previous studies have indicated that frequency–domain analysis parameters are also associated with cardiovascular mortality in heart failure patients [40]. However, in this study, LF, HF, and ULF showed only moderate predictive ability for MACEs and cardiovascular mortality. TP, VLF, and the LF/HF ratio exhibited weaker predictive capabilities. The LF and HF bands primarily reflect changes in sympathetic and parasympathetic nervous system activity. Therefore, the predictive capacity of LF and HF for cardiovascular events reflects the imbalance of the ANS in CHF patients [41]. LF, as an indicator of the joint influence of the sympathetic and parasympathetic nervous systems, typically decreases during sympathetic activation or parasympathetic inhibition [42]. Therefore, a reduction in LF may indicate sympathetic overactivation and parasympathetic dysfunction, which is closely related to cardiac remodeling and the risk of arrhythmias in CHF patients. HF, reflecting parasympathetic regulation, tends to decrease when parasympathetic activity is reduced, further exacerbating pathological cardiac changes and increasing the risk of MACEs and cardiovascular mortality [43]. In contrast, the poorer predictive ability of TP, VLF, ULF, and the LF/HF ratio may be because these frequency bands are influenced by various physiological and pathological factors and may also be confounded by other factors such as medication and emotional fluctuations. Additionally, this discrepancy might arise from differences in the study population, methodology, or the specific endpoints assessed [44, 45]. The duration of HRV monitoring, the devices used for data collection, and the statistical models employed could all impact the results and their interpretation.

## 5. Advantages and Limitations

Currently, there is a lack of research regarding the prognostic significance of 24-h Holter monitoring of HRV in predicting MACEs in CHF patients. This study provides a comprehensive perspective on the prognostic value of various HRV parameters in assessing the outcomes of CHF patients. The multicenter cohort design, which includes patients from different regions of Xinjiang, China, enhances the generalizability and representativeness of the study findings. However, there are certain limitations. As an observational study, potential confounding factors could not be fully controlled, which might affect the interpretation of the results. Additionally, the relatively short follow-up period of the study does not allow for a thorough assessment of long-term out-of-hospital events. Therefore, longer and more detailed follow-up studies are needed in the future. Lastly, while the survey may highly represent the population in Xinjiang, China, the results should be interpreted with caution when applied to other populations, due to differences in lifestyle and environmental factors.

## 6. Conclusion

In conclusion, this study provides valuable evidence for applying HRV as a prognostic tool in CHF patients, highlighting the critical role of ANS dysfunction in the pathophysiology of CHF. The study identifies potential risk prediction markers for clinical use. Specifically, HRV time–domain parameters, including SDNN, triangular index, SDNN index, SDANN index, NN50, and rMSSD, are associated with out-of-hospital MACEs and cardiovascular mortality in CHF patients. Frequency–domain parameters, such as LF, HF, and ULF, are linked to MACEs, though their association with cardiovascular mortality is weaker. Among the HRV parameters, the triangular index demonstrates the best predictive ability for both MACEs and cardiovascular mortality.

## Figures and Tables

**Table 1:** Baseline characteristics of CHF patients grouped by MACEs.

**Characteristics**	**Total (** **n** = 906**)**	**Non-MACEs (** **n** = 695**)**	**MACEs (** **n** = 211**)**	**Statistic**	**p** ** value**
*Demographic information*					
Sex				0.151	0.697
Female	302 (33.3)	234 (33.7)	68 (32.2)		
Male	604 (66.7)	461 (66.3)	143 (67.8)		
Age, years	61.0 (52.0,71.0)	61.0 (51.0,69.0)	65.0 (55.0,74.0)	−4.143	< 0.001
BMI, kg/m^2^	25.1 (23.1, 27.4)	25.3 (23.4, 27.5)	24.8 (22.2, 26.9)	2.512	0.012
SBP, mmHg	122.0 (110.0, 138.0)	122.0 (110.0, 137.0)	121.0 (110.0, 140.0)	−0.51	0.61
DBP, mmHg	76.0 (67.0, 84.0)	75.0 (67.0, 83.0)	76.0 (67.0, 88.0)	−1.416	0.157
Smoking status				17.045	< 0.001
Never	588 (64.9)	474 (68.2)	114 (54.0)		
Former	125 (13.8)	93 (13.4)	32 (15.2)		
Now	193 (21.3)	128 (18.4)	65 (30.8)		
Drinking status				7.352	0.025
Never	767 (84.7)	576 (82.9)	191 (90.5)		
Former	58 (6.4)	49 (7.1)	9 (4.3)		
Now	81 (8.9)	70 (10.1)	11 (5.2)		
Medical condition					
Hypertension	660 (72.8)	491 (70.6)	169 (80.1)	7.304	0.007
Diabetes	306 (33.8)	239 (34.4)	67 (31.8)	0.502	0.478
CHD	590 (65.1)	450 (64.7)	140 (66.4)	0.183	0.669
NYHA classification				36.21	< 0.001
II	176 (19.4)	143 (20.6)	33 (15.6)		
III	538 (59.4)	436 (62.7)	102 (48.3)		
IV	192 (21.2)	116 (16.7)	76 (36.0)		
*Clinical examinations*					
WBC, ×10^9^	7.1 (5.7, 8.7)	7.1 (5.7, 8.6)	6.9 (5.5, 9.0)	−0.155	0.877
Neutrophil, ×10^9^	4.5 (3.5, 6.0)	4.5 (3.4, 5.9)	4.7 (3.7, 6.2)	−1.731	0.084
Lymphocyte, ×10^9^	1.6 (1.2, 2.1)	1.7 (1.3, 2.1)	1.4 (1.0, 1.8)	5.093	< 0.001
Erythrocyte, ×10^6^	4.7 (4.2, 5.1)	4.7 (4.3, 5.1)	4.6 (4.1, 5.0)	1.383	0.167
Hemoglobin, g	138.0 (124.0, 149.0)	139.0 (125.0, 150.0)	136.0 (120.0, 148.0)	1.836	0.066
NT-proBNP, pg/mL	2560 (892, 5843)	2438 (794, 5480)	3173 (1415, 6440)	−2.157	0.031
eGFR, mL/min/1.73 m^2^	86.8 (70.2, 98.8)	87.8 (72.1, 99.8)	82.1 (61.7, 95.4)	3.72	< 0.001
FBG, mmol/L	6.1 (5.2, 7.8)	6.0 (5.2, 7.7)	6.3 (5.3, 8.2)	−1.317	0.188
HbA1c, %	6.2 (5.8, 7.1)	6.2 (5.7, 7.0)	6.3 (5.9, 7.2)	−2.442	0.015
Triglyceride, mmol/L	1.2 (0.9, 1.6)	1.2 (0.9, 1.7)	1.1 (0.9, 1.5)	2.023	0.043
TC, mmol/L	3.8 (3.1, 4.5)	3.8 (3.1, 4.6)	3.7 (3.1, 4.3)	2.011	0.044
HDL-C, mmol/L	0.9 (0.8, 1.1)	0.9 (0.8, 1.1)	0.9 (0.7, 1.0)	2.843	0.004
LDL-C, mmol/L	2.3 (1.8, 2.9)	2.3 (1.9, 3.0)	2.2 (1.8, 2.7)	2.949	0.003
Albumin, g/L	39.2 (35.9, 42.0)	39.6 (36.4, 42.4)	37.9 (33.9, 41.1)	4.475	< 0.001
CRP, mg/L	10.8 (7.2, 17.8)	10.7 (7.1, 16.3)	11.7 (7.5, 23.5)	−1.891	0.059
IL-6, ng/L	8.2 (4.7, 17.4)	7.5 (4.2, 15.3)	12.7 (5.7, 22.8)	−4.923	< 0.001
LVEF, %	42.5 (36.8, 53.2)	42.2 (36.8, 52.3)	43.1 (37.2, 55.9)	−1.513	0.13

*Note:* Continuous variables are expressed as medians (P25, P75) and categorical variables as frequencies (percentages).

Abbreviations: BMI, body mass index; CHD, coronary heart disease; CRP, C-reactive protein; DBP, diastolic blood pressure; eGFR, estimated glomerular filtration rate; FBG, fasting blood glucose; HbA1c, glycated hemoglobin; HDL-C, high-density lipoprotein cholesterol; IL-6, interleukin-6; LDL-C, low-density lipoprotein cholesterol; LVEF, left ventricular ejection fraction; NT-proBNP, N-terminal pro-brain natriuretic peptide; NYHA, New York Heart Association; SBP, systolic blood pressure; TC, total cholesterol; WBC, white blood cell.

**Table 2:** Heart rate variability parameters in CHF patients grouped by MACEs.

**Characteristics**	**Total (** **n** = 906**)**	**Non-MACEs (** **n** = 695**)**	**MACEs (** **n** = 211**)**	**Statistic**	**p** ** value**
Average heart rate	76.0 (68.0, 85.0)	75.0 (68.0, 83.0)	82.0 (71.0, 93.0)	−5.638	< 0.001
Slowest heart rate	58.0 (51.0, 66.0)	57.0 (50.0, 65.0)	64.0 (55.0, 77.0)	−6.952	< 0.001
Fastest heart rate	105.0 (95.0, 117.0)	104.0 (95.0, 115.0)	108.0 (95.0, 121.0)	−2.113	0.035
*Time–domain analysis*					
SDNN, ms	71.5 (53.1, 96.3)	76.1 (57.8, 98.5)	58.9 (34.1, 74.0)	8.770	< 0.001
Triangular index	34.8 (25.4, 48.8)	37.5 (28.6, 52.4)	26.2 (16.2, 37.7)	9.285	< 0.001
SDNN index, ms	17.5 (10.6, 28.7)	18.4 (11.7, 29.7)	13.1 (7.3, 24.0)	5.557	< 0.001
SDANN index, ms	61.0 (45.5, 80.4)	64.7 (48.7, 82.8)	48.0 (30.9, 66.2)	8.520	< 0.001
NN50	1743.0 (550.0, 5023.0)	1900.0 (640.0, 5709.0)	1324.0 (329.0, 3506.0)	4.070	< 0.001
rMSSD, ms	21.5 (15.1, 32.6)	22.3 (16.0, 34.1)	19.1 (12.8, 26.0)	5.047	< 0.001
pNN50, %	9.7 (3.2, 28.0)	9.5 (3.3, 28.0)	10.0 (3.0, 28.0)	0.176	0.86
*Frequency–domain analysis*					
Total power, ms^2^	1950.8 (700.4, 5830.3)	2277.9 (786.1, 5963.3)	1471.3 (435.8, 4904.0)	3.270	0.001
ULF, ms^2^	765.2 (159.6, 3277.2)	882.0 (188.7, 3334.0)	548.5 (83.7, 2779.3)	2.327	0.02
VLF, ms^2^	1032.4 (360.0, 2560.3)	1094.3 (405.7, 2721.4)	752.5 (228.5, 1995.0)	3.293	< 0.001
LF, ms^2^	89.0 (33.1, 223.6)	103.3 (41.6, 241.7)	54.1 (15.2, 127.0)	5.463	< 0.001
HF, ms^2^	42.8 (19.2, 107.1)	47.9 (21.8, 121.6)	31.3 (13.0, 58.7)	4.982	< 0.001
LF/HF	2.1 (1.0, 3.5)	2.1 (1.0, 3.6)	2.1 (1.0, 3.3)	0.906	0.365

Abbreviations: HF, high frequency; LF, low frequency; NN50, number of pairs of adjacent normal-to-normal intervals differing by more than 50 ms; pNN50, percentage of adjacent normal-to-normal intervals differing by more than 50 ms; rMSSD, root mean square of successive differences between adjacent normal-to-normal intervals; SDANN index, standard deviation of the averages of normal-to-normal intervals for each 5-min segment; SDNN, standard deviation of all normal-to-normal intervals; SDNN index, mean of the standard deviations of all normal-to-normal intervals for each 5-min segment; ULF, ultralow frequency; VLF, very low frequency.

**Table 3:** Cox proportional risk regression analysis of heart rate variability parameters and incidence of MACEs in patients with CHF.

**Characteristics**	**Crude model**		**Model 1**		**Model 2**	
	**HR (95% CI)**	**p** ** value**	**HR (95% CI)**	**p** ** value**	**HR (95% CI)**	**p** ** value**
*Time–domain analysis*						
SDNN	0.974 (0.968, 0.980)	< 0.001	0.976 (0.970, 0.982)	< 0.001	0.976 (0.970, 0.981)	< 0.001
Triangular index	0.958 (0.948, 0.967)	< 0.001	0.962 (0.952, 0.972)	< 0.001	0.963 (0.953, 0.973)	< 0.001
SDNN index	0.983 (0.974, 0.992)	< 0.001	0.982 (0.973, 0.991)	< 0.001	0.983 (0.974, 0.992)	< 0.001
SDANN index	0.973 (0.966, 0.979)	< 0.001	0.975 (0.969, 0.982)	< 0.001	0.974 (0.967, 0.981)	< 0.001
NN50 (log)	0.839 (0.771, 0.912)	< 0.001	0.840 (0.772, 0.914)	< 0.001	0.859 (0.787, 0.937)	< 0.001
rMSSD	0.980 (0.971, 0.990)	< 0.001	0.979 (0.969, 0.989)	< 0.001	0.980 (0.970, 0.989)	< 0.001
pNN50	0.999 (0.993, 1.004)	0.628	0.998 (0.992, 1.003)	0.414	0.996 (0.990, 1.002)	0.186
*Frequency–domain analysis*						
TP (log)	0.861 (0.790, 0.939)	< 0.001	0.870 (0.798, 0.948)	0.002	0.890 (0.816, 0.971)	0.009
ULF (log)	0.931 (0.876, 0.989)	0.021	0.936 (0.880, 0.995)	0.035	0.945 (0.888, 1.006)	0.079
VLF (log)	0.858 (0.790, 0.932)	< 0.001	0.871 (0.802, 0.947)	0.001	0.889 (0.815, 0.969)	0.007
LF (log)	0.780 (0.712, 0.855)	< 0.001	0.802 (0.733, 0.877)	< 0.001	0.817 (0.743, 0.898)	< 0.001
HF (log)	0.787 (0.711, 0.872)	< 0.001	0.784 (0.708, 0.869)	< 0.001	0.806 (0.728, 0.893)	< 0.001
LF/HF	0.965 (0.913, 1.020)	0.211	0.977 (0.930, 1.027)	0.359	0.981 (0.936, 1.028)	0.426

*Note:* “log” is a variable that is put through a logarithmic transformation with a natural constant (*e*) as the base. The crude model was unadjusted for any covariates. Model 1 was adjusted for sex, age, BMI, smoking status, drinking status, hypertension, and NYHA classification. Model 2 included further adjustments for laboratory markers based on Model 1, incorporating lymphocyte count, eGFR, NT-proBNP, albumin, HbA1c, TG, TC, HDL-C, LDL-C, and IL-6.

Abbreviations: 95% CI, 95% confidence interval; HR, hazard ratio.

**Table 4:** Cox proportional risk regression analysis of heart rate variability parameters and incidence of cardiac death in patients with CHF.

**Characteristics**	**Crude model**		**Model 1**		**Model 2**	
	**HR (95% CI)**	**p** ** value**	**HR (95% CI)**	**p** ** value**	**HR (95% CI)**	**p** ** value**
*Time–domain analysis*						
SDNN	0.968 (0.953, 0.983)	< 0.001	0.973 (0.959, 0.987)	< 0.001	0.973 (0.958, 0.988)	< 0.001
Triangular index	0.908 (0.879, 0.937)	< 0.001	0.918 (0.888, 0.948)	< 0.001	0.913 (0.879, 0.947)	< 0.001
SDNN index	0.988 (0.968, 1.009)	0.269	0.989 (0.970, 1.009)	0.273	0.991 (0.972, 1.011)	0.387
SDANN index	0.965 (0.949, 0.982)	< 0.001	0.971 (0.955, 0.987)	< 0.001	0.969 (0.951, 0.987)	0.001
NN50 (log)	0.982 (0.796, 1.212)	0.865	1.000 (0.811, 1.233)	0.999	1.011 (0.818, 1.249)	0.920
rMSSD	0.970 (0.942, 0.999)	0.043	0.971 (0.944, 0.999)	0.042	0.970 (0.942, 0.998)	0.036
pNN50	1.011 (1.000, 1.023)	0.061	1.009 (0.997, 1.021)	0.128	1.009 (0.996, 1.022)	0.173
*Frequency–domain analysis*						
TP (log)	0.807 (0.657, 0.991)	0.041	0.840 (0.683, 1.033)	0.098	0.894 (0.724, 1.103)	0.296
ULF (log)	0.818 (0.713, 0.939)	0.004	0.833 (0.723, 0.960)	0.011	0.866 (0.745, 1.007)	0.061
VLF (log)	0.803 (0.664, 0.970)	0.023	0.832 (0.686, 1.009)	0.062	0.881 (0.716, 1.085)	0.234
LF (log)	0.707 (0.570, 0.877)	0.002	0.753 (0.610, 0.931)	0.009	0.804 (0.639, 1.011)	0.062
HF (log)	0.758 (0.591, 0.973)	0.03	0.787 (0.617, 1.003)	0.053	0.853 (0.680, 1.070)	0.169
LF/HF	0.824 (0.668, 1.016)	0.069	0.842 (0.679, 1.045)	0.119	0.882 (0.716, 1.088)	0.242

*Note:* “log” is a variable put through a logarithmic transformation with a natural constant (*e*) as the base. The crude model was unadjusted for any covariates. Model 1 was adjusted for sex, age, BMI, smoking status, drinking status, hypertension, and NYHA classification. Model 2 included further adjustments for laboratory markers based on Model 1, incorporating lymphocyte count, eGFR, NT-proBNP, albumin, HbA1c, TG, TC, HDL-C, LDL-C, and IL-6.

Abbreviations: CI, confidence interval; HR, hazard ratio.

**Table 5:** ROC analysis of heart rate variability parameters and occurrence of MACEs in patients with CHF.

**Characteristics**	**AUC (95% CI)**	**Sensitivity**	**Specificity**	**Cut-off value**	**Youden index**
*Time–domain analysis*					
SDNN	0.699 (0.655–0.743)	0.365	0.991	38.390	0.356
Triangular index	0.711 (0.668–0.753)	0.474	0.879	22.745	0.353
SDNN index	0.626 (0.580–0.672)	0.336	0.894	8.515	0.230
SDANN index	0.693 (0.650–0.737)	0.365	0.942	37.020	0.307
NN50 (log)	0.592 (0.548–0.636)	0.374	0.764	6.404	0.138
rMSSD	0.615 (0.571–0.658)	0.791	0.390	27.280	0.181
pNN50	0.496 (0.451–0.541)	0.464	0.571	11.935	0.036
*Frequency–domain analysis*					
TP (log)	0.574 (0.528–0.620)	0.280	0.858	6.148	0.137
ULF (log)	0.553 (0.507–0.598)	0.446	0.665	5.775	0.110
VLF (log)	0.575 (0.530–0.620)	0.441	0.689	6.330	0.130
LF (log)	0.624 (0.578–0.670)	0.427	0.787	3.561	0.214
HF (log)	0.613 (0.569–0.657)	0.768	0.426	4.136	0.194
LF/HF	0.521 (0.477–0.564)	0.900	0.164	4.365	0.065

*Note:* “log” is a variable put through a logarithmic transformation with a natural constant (*e*) as the base.

Abbreviations: AUC, area under the curve; CI, confidence interval.

**Table 6:** Incremental predictive value and predictive power of each HRV metric in predicting MACEs in models evaluated using the NRI, IDI, and *C*-index.

**Characteristics**	**C** **-index**	**p** ** value**	**IDI**	**p** ** value**	**Continuous NRI**	**p** ** value**
Original model	0.785 (0.703–0.866)	< 0.001	Ref.		Ref.	
*Time*–*domain analysis*						
+SDNN	0.817 (0.744–0.891)	< 0.001	0.098 (0.055–0.147)	< 0.001	0.271 (0.153–0.342)	< 0.001
+Triangular index	0.850 (0.794–0.907)	< 0.001	0.075 (0.034–0.124)	< 0.001	0.195 (0.106–0.311)	0.002
+SDNN index	0.795 (0.721–0.868)	< 0.001	0.022 (0.006–0.048)	< 0.001	0.132 (0.033–0.216)	0.014
+SDANN index	0.812 (0.740–0.883)	< 0.001	0.081 (0.040–0.126)	< 0.001	0.230 (0.118–0.308)	< 0.001
+NN50 (log)	0.781 (0.705–0.856)	< 0.001	0.016 (0.003–0.041)	0.006	0.138 (0.025–0.227)	0.020
+rMSSD	0.811 (0.737–0.886)	< 0.001	0.037 (0.014–0.063)	< 0.001	0.190 (0.091–0.289)	0.002
+pNN50	0.780 (0.699–0.862)	< 0.001	0.001(−0.001-0.008)	0.368	−0.021 (−0.102 to 0.094)	0.885
*Frequency*–*domain analysis*						
+TP (log)	0.786 (0.708–0.864)	< 0.001	0.014 (0.003–0.038)	0.002	0.090 (−0.012–0.195)	0.066
+ULF (log)	0.788 (0.709–0.867)	< 0.001	0.007 (0.000–0.024)	0.034	0.090 (−0.045 to 0.174)	0.172
+VLF (log)	0.788 (0.710–0.865)	< 0.001	0.013 (0.002–0.034)	0.01	0.068 (−0.032 to 0.154)	0.164
+LF (log)	0.794 (0.715–0.873)	< 0.001	0.028 (0.009–0.057)	< 0.001	0.134 (0.034–0.223)	0.018
+HF (log)	0.783 (0.698–0.868)	< 0.001	0.029 (0.011–0.057)	< 0.001	0.183 (0.096–0.274)	0.002
+LF/HF	0.787 (0.706–0.868)	< 0.001	0.001 (−0.001-0.006)	0.374	−0.002 (−0.101 to 0.122)	0.810

*Note:* “Original model” refers to the joint creation of a proportional risk regression model for Cox using gender, age, body mass index, smoking status, drinking status, hypertension, NYHA classification, lymphocyte count, eGFR, NT-proBNP, albumin, HbA1c, TG, TC, HDL-C, LDL-C, and IL-6. “log” is a variable put through a logarithmic transformation with a natural constant (*e*) as the base.

Abbreviations: *C*-index, concordance index; IDI, integrated discrimination improvement; NRI, net reclassification improvement.

## Data Availability

The datasets generated during and/or analyzed during the current study are available from the corresponding author upon reasonable request.

## Nomenclature

CHF: chronic heart failure
ANS: autonomic nervous system
HRV: heart rate variability
MACEs: major adverse cardiovascular events
NT-proBNP: N-terminal pro-B-type natriuretic peptide
HbA1c: hemoglobin A1c
HDL-C: high-density lipoprotein cholesterol
CRP: C-reactive protein
IL-6: interleukin-6
SDNN: standard deviation of all normal-to-normal intervals
SDANN: standard deviation of the averages of NN intervals
NN50: number of pairs of successive NN intervals that differ by more than 50 ms
rMSSD: root mean square of successive differences between adjacent NN intervals
TP: total power
LF: low frequency
HF: high frequency
ULF: ultralow frequency
VLF: very low frequency

## Supporting Information

Supporting Information 1 Figure S1: Survival curves for the incidence of cardiovascular death in CHF patients grouped by tertiles of each HRV time–domain analysis metric.

Supporting Information 2 Figure S2: Survival curves for the incidence of cardiovascular death in CHF patients grouped by tertiles of each HRV frequency–domain analysis metric.

Supporting Information 3 Table S1: Spearman's correlation coefficient and *p* value of HRV index with cardiac risk factors. Table S2: ROC analysis of heart rate variability parameters and occurrence of cardiac death in patients with CHF. Table S3: Incremental predictive value and predictive power of each HRV metric in predicting cardiac death in models evaluated using the NRI, IDI, and *C*-index.
