# Supplementary figures and images for: Heart Rate Variability's Value in Predicting Out-of-Hospital Major Adverse Cardiovascular Events in Patients With Chronic Heart Failure
Source: Cardiovasc Ther. 2025 Aug 14;2025:6412775. doi: 10.1155/cdr/6412775 (PMC12370394; doi:10.1155/cdr/6412775)

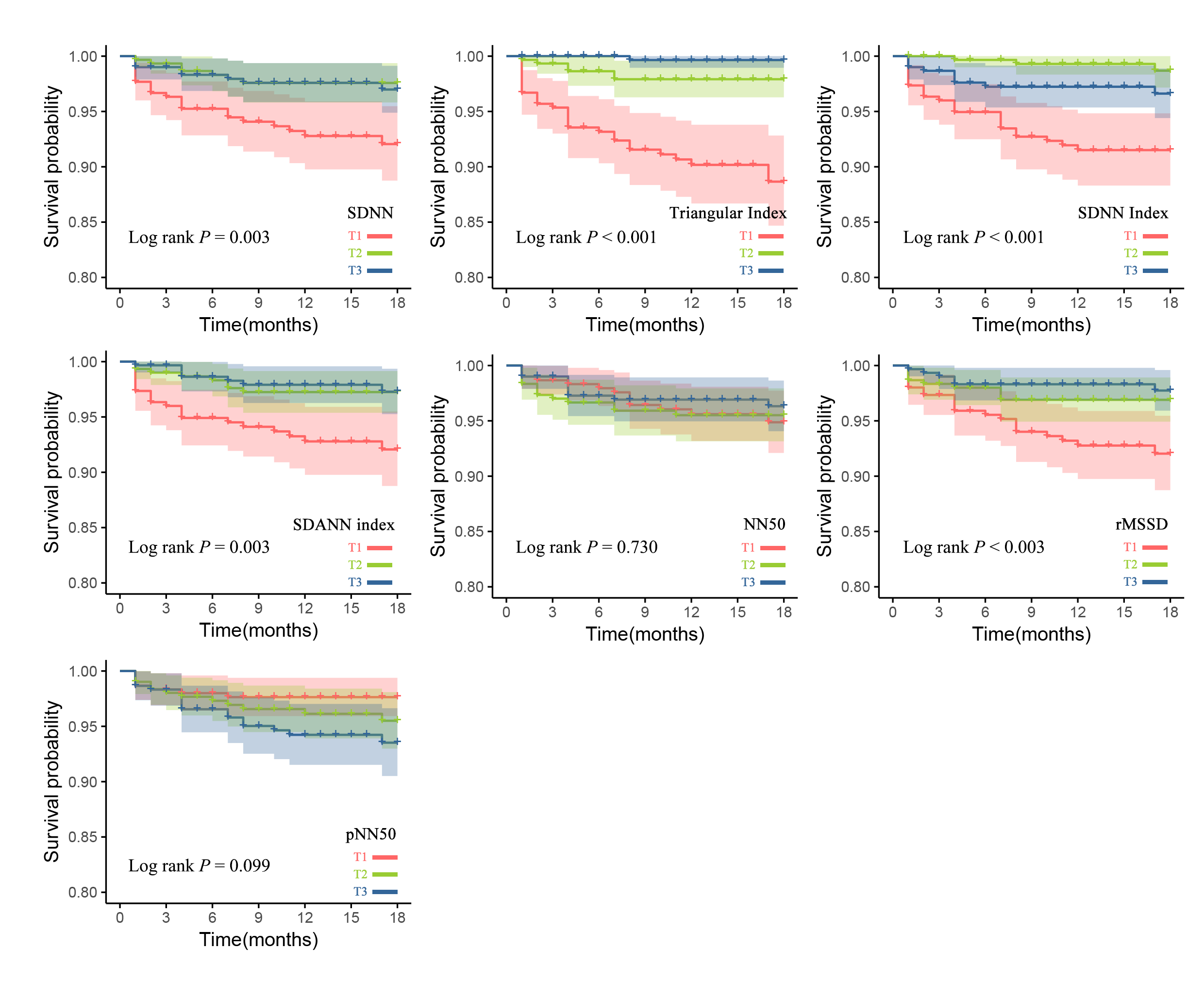

Supplement: Supporting Information 1 — Figure S1: Survival curves for the incidence of cardiovascular death in CHF patients grouped by tertiles of each HRV time–domain analysis metric. [file 6412775.f1.tiff]

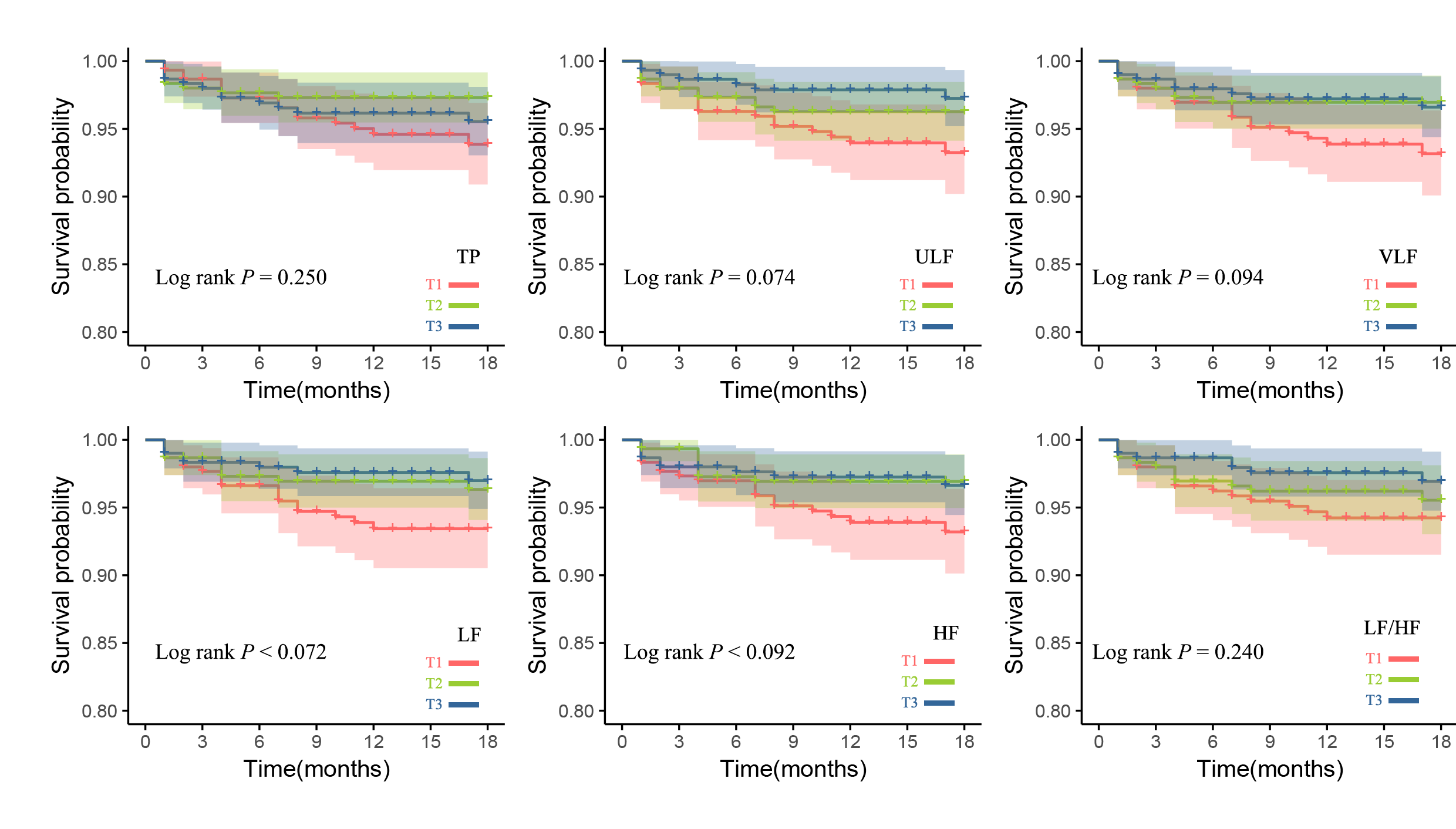

Supplement: Supporting Information 2 — Figure S2: Survival curves for the incidence of cardiovascular death in CHF patients grouped by tertiles of each HRV frequency–domain analysis metric. [file 6412775.f2.tiff]
