# Supplementary material for: Heart Rate Variability's Value in Predicting Out-of-Hospital Major Adverse Cardiovascular Events in Patients With Chronic Heart Failure
Source: Cardiovasc Ther. 2025 Aug 14;2025:6412775. doi: 10.1155/cdr/6412775 (PMC12370394; doi:10.1155/cdr/6412775)
Supplement: Supporting Information 3 — Table S1: Spearman's correlation coefficient and p value of HRV index with cardiac risk factors. Table S2: ROC analysis of heart rate variability parameters and occurrence of cardiac death in patients with CHF. Table S3: Incremental predictive value and predictive power of each HRV metric in predicting cardiac death in models evaluated using the NRI, IDI, and C-index. [file 6412775.f3.docx]

Table S1: Spearman's correlation coefficient and p-value of HRV index with cardiac risk factors.

| **Characteristics** | **SDNN** | **Trigonometric index** | **SDNN index** | **SDANN index** | **NN50** | **rMSSD** | **pNN50** | **TP** | **ULF** | **VLF** | **LF** | **HF** | **LF:HF** |
| --- | --- | --- | --- | --- | --- | --- | --- | --- | --- | --- | --- | --- | --- |
| Age | 0.01 | -0.06 | 0.06 | -0.02 | 0.06 | 0.10 | 0.01 | -0.01 | 0.00 | -0.05 | -0.07 | 0.07 | -0.19 |
| BMI | -0.03 | 0.00 | 0.05 | -0.02 | 0.04 | 0.07 | -0.06 | 0.01 | -0.01 | 0.01 | 0.04 | 0.03 | 0.01 |
| NT proBNP | -0.29 | -0.30 | -0.23 | -0.27 | -0.14 | -0.16 | 0.01 | -0.19 | -0.17 | -0.17 | -0.25 | -0.20 | -0.09 |
| eGFR | 0.11 | 0.12 | 0.05 | 0.12 | 0.10 | 0.05 | 0.01 | 0.05 | 0.02 | 0.06 | 0.11 | 0.06 | 0.06 |
| HbA1c | -0.21 | -0.19 | -0.20 | -0.18 | -0.15 | -0.15 | 0.11 | -0.08 | -0.05 | -0.11 | -0.17 | -0.14 | -0.04 |
| TG | -0.05 | 0.01 | -0.05 | -0.02 | -0.06 | -0.10 | -0.04 | -0.01 | 0.00 | -0.01 | -0.01 | -0.04 | 0.05 |
| TC | 0.08 | 0.10 | 0.06 | 0.11 | 0.02 | -0.01 | -0.04 | 0.00 | 0.00 | 0.01 | 0.04 | 0.03 | 0.03 |
| HDL-C | 0.16 | 0.16 | 0.17 | 0.16 | 0.13 | 0.15 | -0.06 | 0.05 | 0.03 | 0.06 | 0.12 | 0.15 | -0.03 |
| LDL-C | 0.07 | 0.09 | 0.04 | 0.10 | 0.00 | -0.03 | -0.04 | 0.00 | -0.02 | 0.00 | 0.04 | 0.01 | 0.05 |
| LVEF | 0.06 | 0.04 | 0.05 | 0.06 | 0.08 | 0.09 | 0.01 | 0.09 | 0.08 | 0.03 | 0.06 | 0.10 | -0.06 |
| IL-6 | -0.22 | -0.31 | -0.12 | -0.23 | -0.09 | -0.08 | 0.04 | -0.09 | -0.08 | -0.13 | -0.19 | -0.11 | -0.14 |
| CRP | -0.16 | -0.18 | -0.11 | -0.16 | -0.06 | -0.08 | 0.03 | -0.10 | -0.09 | -0.13 | -0.14 | -0.12 | -0.04 |
| **P-value** | **SDNN** | **Trigonometric index** | **SDNN index** | **SDANN index** | **NN50** | **rMSSD** | **pNN50** | **TP** | **ULF** | **VLF** | **LF** | **HF** | **LF:HF** |
| P for Age | 0.838 | 0.053 | 0.063 | 0.618 | 0.070 | **0.004** | 0.695 | 0.753 | 0.986 | 0.107 | **0.035** | **0.040** | **<0.001** |
| P for BMI | 0.387 | 0.894 | 0.136 | 0.617 | 0.249 | **0.037** | 0.051 | 0.851 | 0.714 | 0.765 | 0.280 | 0.411 | 0.749 |
| P for NT proBNP | **<0.001** | **<0.001** | **<0.001** | **<0.001** | **<0.001** | **<0.001** | 0.750 | **<0.001** | **<0.001** | **<0.001** | **<0.001** | **<0.001** | **0.006** |
| P for eGFR | **0.001** | **<0.001** | 0.141 | **0.001** | **0.003** | 0.162 | 0.763 | 0.140 | 0.616 | 0.060 | **0.001** | 0.062 | 0.062 |
| P for HbA1c | **<0.001** | **<0.001** | **<0.001** | **<0.001** | **<0.001** | **<0.001** | **0.001** | **0.018** | 0.169 | **0.001** | **<0.001** | **<0.001** | 0.183 |
| P for TG | 0.120 | 0.871 | 0.150 | 0.597 | 0.087 | **0.004** | 0.270 | 0.716 | 0.987 | 0.664 | 0.768 | 0.194 | 0.099 |
| P for TC | **0.014** | **0.004** | 0.058 | **0.001** | 0.542 | 0.800 | 0.191 | 0.906 | 0.887 | 0.817 | 0.249 | 0.318 | 0.353 |
| P for HDL-C | **<0.001** | **<0.001** | **<0.001** | **<0.001** | **<0.001** | **<0.001** | 0.053 | 0.150 | 0.294 | 0.078 | **<0.001** | **<0.001** | 0.399 |
| P for LDL-C | **0.036** | **0.006** | 0.253 | **0.003** | 0.910 | 0.359 | 0.180 | 0.902 | 0.622 | 0.986 | 0.258 | 0.734 | 0.154 |
| P for LVEF | 0.053 | 0.224 | 0.166 | 0.054 | **0.022** | **0.006** | 0.787 | **0.007** | **0.019** | 0.396 | 0.097 | **0.003** | 0.052 |
| P for IL-6 | **<0.001** | **<0.001** | **<0.001** | **<0.001** | **0.008** | **0.012** | 0.211 | **0.005** | **0.021** | **<0.001** | **<0.001** | **0.001** | **<0.001** |
| P for CRP | **<0.001** | **<0.001** | **0.001** | **<0.001** | 0.075 | **0.022** | 0.324 | **0.004** | **0.010** | **<0.001** | **<0.001** | **<0.001** | 0.248 |

Abbreviations: BMI: body mass index; NT-proBNP: N-terminal pro-brain natriuretic peptide; eGFR: estimated glomerular filtration rate; FBG: fasting blood glucose; HbA1c: glycated hemoglobin; TC: total cholesterol; HDL-C: high-density lipoprotein cholesterol; LDL-C: low-density lipoprotein cholesterol; CRP: C-reactive protein; IL-6: interleukin-6;

Table S2: ROC analysis of heart rate variability parameters and occurrence of cardiac death in patients with CHF.

| **Characteristics** | **AUC (95%CI)** | **Sensitivity** | **Specificity** | **Cut-off value** | **Youden index** |
| --- | --- | --- | --- | --- | --- |
| **Time-Domain Analysis** | |  |  |  |  |
| SDNN | 0.691(0.577 – 0.804) | 0.556 | 0.840 | 48.180 | 0.396 |
| Triangular Index | 0.807(0.741 – 0.874) | 0.806 | 0.706 | 28.315 | 0.511 |
| SDNN index | 0.670(0.556 – 0.784) | 0.667 | 0.706 | 12.010 | 0.372 |
| SDANN index | 0.685(0.575 – 0.795) | 0.500 | 0.886 | 37.020 | 0.386 |
| NN50 (log) | 0.522(0.419 – 0.625) | 0.583 | 0.548 | 7.310 | 0.132 |
| rMSSD | 0.650(0.552 – 0.748) | 0.583 | 0.734 | 15.815 | 0.318 |
| pNN50 | 0.605(0.513 – 0.697) | 0.694 | 0.491 | 8.990 | 0.185 |
| **Frequency-Domain Analysis** | |  |  |  |  |
| TP (log) | 0.583(0.478 – 0.688) | 0.417 | 0.772 | 6.455 | 0.189 |
| ULF (log) | 0.636(0.541 – 0.730) | 0.778 | 0.491 | 6.786 | 0.269 |
| VLF (log) | 0.598(0.503 – 0.694) | 0.556 | 0.643 | 6.422 | 0.198 |
| LF (log) | 0.640(0.534 – 0.746) | 0.722 | 0.570 | 4.270 | 0.292 |
| HF (log) | 0.605(0.495 – 0.716) | 0.389 | 0.820 | 2.688 | 0.208 |
| LF/HF | 0.593(0.500 – 0.685) | 0.639 | 0.548 | 1.905 | 0.187 |

Abbreviations: AUC: Area Under the Curve; CI: confidence interval.

Note: “log” is a variable put through a logarithmic transformation with a natural constant (e) as the base.

Table S3: Incremental predictive value and predictive power of each HRV metric in predicting cardiac death in models evaluated using the NRI, IDI, and C-index.

| **Characteristics** | C-index | *P*-value | IDI | *P*-value | Continuous NRI | *P*-value |
| --- | --- | --- | --- | --- | --- | --- |
| **Original model** | 0.838(0.778-0.898) | <0.001 | Ref. |  | Ref. |  |
| **Time-Domain Analysis** |  |  |  |  |  |  |
| SDNN | 0.866(0.815-0.918) | <0.001 | 0.025(-0.005-0.176) | 0.104 | 0.268(-0.064-0.465) | 0.076 |
| Triangular Index | 0.891(0.851-0.930) | <0.001 | 0.087(0.035-0.205) | <0.001 | 0.420(0.192-0.581) | <0.001 |
| SDNN index | 0.845(0.789-0.901) | <0.001 | -0.005(-0.009-0.055) | 0.271 | 0.207(-0.247-0.354) | 0.462 |
| SDANN index | 0.862(0.808-0.916) | <0.001 | 0.028(-0.001-0.154) | 0.056 | 0.221(-0.053-0.422) | 0.082 |
| NN50 (log) | 0.838(0.777-0.898) | <0.001 | 0.002(-0.008-0.060) | 0.685 | -0.083(-0.206-0.264) | 0.177 |
| rMSSD | 0.861(0.809-0.914) | <0.001 | -0.005(-0.011-0.059) | 0.634 | 0.355(0.037-0.471) | 0.032 |
| pNN50 | 0.840(0.782-0.899) | <0.001 | 0.006(-0.003-0.064) | 0.266 | 0.184(-0.174-0.354) | 0.262 |
| **Frequency-Domain Analysis** |  |  |  |  |  |  |
| TP (log) | 0.844(0.789-0.899) | <0.001 | -0.001(-0.009-0.049) | 0.327 | 0.102(-0.181-0.289) | 0.454 |
| ULF (log) | 0.846(0.792-0.900) | <0.001 | 0.004(-0.009-0.058) | 0.452 | 0.228(-0.131-0.354) | 0.216 |
| VLF (log) | 0.848(0.795-0.902) | <0.001 | -0.002(-0.011-0.046) | 0.183 | 0.131(-0.223-0.316) | 0.422 |
| LF (log) | 0.852(0.800-0.904) | <0.001 | 0.000(-0.013-0.076) | 0.465 | 0.137(-0.151-0.343) | 0.276 |
| HF (log) | 0.842(0.784-0.899) | <0.001 | 0.001(-0.008-0.061) | 0.44 | 0.093(-0.177-0.288) | 0.29 |
| LF/HF | 0.848(0.795-0.901) | <0.001 | 0.001(-0.006-0.038) | 0.43 | 0.121(-0.184-0.282) | 0.358 |

Abbreviations: C-index: concordance index; IDI: integrated discrimination improvement; NRI: net reclassification improvement.

Note: “log” is a variable put through a logarithmic transformation with a natural constant (e) as the base.
